# Association between 4-dimension lifestyle pattern and 10-year mortality risk in Chinese individuals older than 65: a population-based cohort study

**DOI:** 10.18632/aging.202695

**Published:** 2021-03-19

**Authors:** Guangqi Liu, Zheng Xie, Yuanjie Pang, Tao Huang, Yangmu Huang

**Affiliations:** 1 Department of Global Health, Peking University School of Public Health, Haidian 100191, Beijing, China; 2 Institute for Global Health, Peking University, Haidian 100191, Beijing, China; 3 Department of Epidemiology and Biostatistics, Peking University School of Public Health, Haidian 100191, Beijing, China

**Keywords:** lifestyle, mortality, elderly, cohort studies, Chinese

## Abstract

While the impact of a 4-dimension lifestyle pattern (4DL) on older people’s mortality (aged ≥65 years) has been reported in high-income countries, few studies investigated the association between lifestyle pattern and disease-accompanied mortality, or examined the difference among different age or gender groups in low- and middle-income countries. We followed up 16,954 Chinese older participants from 2008 to 2018 and adopted the Cox proportional hazard model to evaluate the protective effect of 4DL. After adjustment for confounders, individuals with 3-4 4DL scores had a 38% reduction in all-cause mortality risk, and up to 36%, 42% and 41% reduced risk of mortality accompanied by hypertension, respiratory disease and dementia, respectively in contrast with those scored 0. Compared with octogenarians, nonagenarians, and centenarians, adhering to 3-4 4DL could further reduce the mortality risks in the younger elderly (aged 65-79 years). This study shows that among the elderly population in China, participants who adhered to 4DL had a lower all-cause mortality risk than those who did not. Additionally, hypertension, respiratory disease, or dementia accompanied mortality risk was also reduced significantly. The findings indicated that the positive effects of 4DL on longevity should be acknowledged in China’s older population, especially for the younger elderly.

## INTRODUCTION

With the increasing life expectancy and falling fertility rates, the aging problem has posed more challenges to global public health [1]. Keeping the older population healthy has been prioritized to lighten the social burden and maintain life quality of older population [2].

Prior research has indicated that lifestyle factors including dietary patterns, smoking status, alcohol consumption and physical activity are long-term factors connected with non-communicable disease prevention and mortality [3, 4]. Due to the interaction among different lifestyles, many studies focused on the relationship between multiple lifestyle factors and health [5–8]. A 4-dimension lifestyle pattern (4DL), which was composed of a healthy diet, non-smoking, moderate alcohol intake and regular physical activity, was applied to assess individual’s health level in many randomized control trials [9, 10]. A significant association was found between healthy lifestyle pattern and lower mortality risk in several European populations. For example, a Swedish prospective cohorts study indicated that adhering to 4DL was associated with more than 50% lower all-cause mortality [11, 12]. While much has been done to explore lifestyle factors in high-income countries, only a few studies were in low- and middle-income countries [5, 13, 14].

Moreover, several studies explored the association between combined effects of healthy lifestyle behaviors and cause-specified mortality, such as diabetes, cardiovascular disease, cancer and heart disease [15–18]. However, few studies examined the associations between 4DL and mortality accompanied by certain diseases, including respiratory diseases, dementia, hypertension, etc. Since older individuals in different age and sex groups have different risk of morbidity and mortality [19], whether the association between 4DL and mortality risks differ across age/gender groups remains unclear.

Therefore, our study aims to construct a modified 4DL and investigate the association between 4DL and 10-year all-cause and disease-accompanied mortality in the older adults from the Chinese Longitudinal Healthy longevity Survey (CLHLS) project. We also conducted subgroup analysis among different age and sex groups. Our study would indicate whether adhering to 4DL leads to changes in mortality among different groups of the older population and provide evidence for policy or lifestyle suggestions for the older population.

## RESULTS

### Basic characteristics of participants

The analyses were based on 6,891 (42.3%) men and 9,389 (57.7%) women who attended the 10-year longitudinal study and provided information on 4DL. Table 1 shows the baseline characteristics of participants subdivided by their 4DL score. In total there were 78834.6 person-years during the 10-year follow up. Overall, 3,954 (33.3%) men and 5,620 (47.3%) women died among the 16,280 participants. The 10-year all-cause mortality rate was 80.6%, and these include 1,285 accompanied by hypertension, 262 with diabetes, 1,071 with heart disease (HD), 1,010 with cerebrovascular disease (CVD), 1,043 with respiratory disease (RD) and 524 with dementia. The rate of loss to follow-up was 27.0%.

**Table 1:** Characteristics of Chinese older adults by 4DL score during 10-year follow-up*.

**Characteristics**	**0 (1,754)**	**1 (7,281)**	**2 (5,543)**	**3-4 (1,702)**	**Total (16,280)**	**P-value**
Sex (%)						<0.001
male	75.0	37.2	38.4	43.7	42.3	
female	25.0	62.8	61.6	56.3	57.7	
Age, mean (SD), y	86.3 (10.6)	88.8 (11.2)	87.1 (11.4)	84.5 (11.7)	87.5 (11.3)	<0.001
Education, mean (SD), y	2.3 (3.3)	1.5 (2.9)	2.1 (3.6)	3.2 (4.3)	2.0 (3.4)	<0.001
Marital status (%)						<0.001
In marriage	40.3	27.1	31.4	40.0	31.3	
Not in marriage	59.7	72.9	68.6	60.0	68.7	
Residence (%)						<0.001
Urban or town	34.1	32.0	44.5	61.5	39.6	
Rural	65.9	68.0	55.5	38.5	60.4	
Economic income (%)						<0.001
High	10.7	11.5	14.4	20.2	13.3	
Medium	69.0	67.2	69.7	69.7	68.5	
Low	20.3	21.3	15.9	10.2	18.2	
BMI, mean (SD)	20.0 (3.1)	19.8(3.1)	20.3(3.3)	21.1(3.3)	20.1(3.2)	
Survival status (%)						<0.001
0	17.0	17.2	21.4	26.9	19.4	
1 (decedent)	83.0	82.8	78.6	73.1	80.6	
Hypertension-accompanied mortality (%)						<0.01
0	86.3	88.0	88.3	90.1	88.2	
1 (decedent)	13.7	12.0	11.7	9.9	11.8	
Diabetes-accompanied mortality (%)						0.89
0	97.9	97.7	97.7	98.0	97.8	
1 (decedent)	2.1	2.3	2.3	2.0	2.2	
HD-accompanied mortality (%)						<0.05
0	90.2	92.2	91.2	91.9	91.6	
1 (decedent)	9.8	7.8	8.8	8.1	8.4	
CVD-accompanied mortality (%)						<0.01
0	91.0	92.4	92.9	94.0	92.6	
1 (decedent)	9.0	7.6	7.1	6.0	7.4	
RD-accompanied mortality (%)						<0.001
0	87.2	91.6	91.9	93.5	91.4	
1 (decedent)	12.8	8.4	8.1	6.5	8.6	
Dementia-accompanied mortality (%)						<0.01
0	95.8	96.3	97.0	97.4	96.6	
1 (decedent)	4.2	3.7	3.0	2.6	3.4	

*Data are expressed as percentage: n (%).

Abbreviations: SD: standard deviation; BMI: body mass index; HD: heart disease; CVD: cerebrovascular disease; RD: respiratory disease.

The mean age of all participants was 87.5 years old, and the average education year was 2.0 years. We found a significant correlation between the proportion of gender and 4DL score. Table 1 shows that participant with 0 4DL had a higher proportion of man compared with other scores. The highest 4DL score participants were more likely to be younger, more educated and live in urban or town. Compared with 0 4DL, individuals with 3-4 4DL scores had a significantly lower probability of accompanying hypertension, heart disease, cardiovascular disease, respiratory disease and dementia when they died.

### Association between 4DL and risk of all-cause mortality

The results of two Cox models are presented in Table 2. After adjusting for age and sex, we found adhering to 4DL was associated with lower mortality rates from all causes during the 10-year follow-up. This disparity in outcomes persisted after multivariable adjustment in model 2. The model 2 was adjusted for sex, age, marital status, educational background, residence, economic income and body mass index (BMI). Compared with those with 0 4DL score, individuals with more scores had a lower risk of mortality. Individuals with 3-4 4DL scores had a 38% reduction in all-cause mortality than those who were scored 0 [HR: 0.72 (0.66-0.79), P<0.001].

**Table 2:** Age and sex adjusted and multivariable adjusted HRs (95% CIs) for all-cause mortality by 4DL score among Chinese older people.

**4DL score**	**Participants**	**Death**	**Model 1**				**Model 2**		
			**Age and sex** **adjusted HR** **(95% CI)**	**P value**	**P for trend**		**Multivariable** **adjusted HR** **(95% CI)**	**P value**	**P for trend**
0	1,754	1,157	1.00 (reference)		<0.001		1.00 (reference)		<0.001
1	7,281	4,568	0.84 (0.79-0.90)	<0.001			0.85 (0.80-0.91)	<0.001	
2	5,543	3,059	0.73 (0.68-0.78)	<0.001			0.76 (0.71-0.82)	<0.001	
3-4	1,702	790	0.65 (0.60-0.72)	<0.001			0.72 (0.66-0.79)	<0.001	

*Model 1 was only adjusted age (as linear term), and sex. Model 2 was further adjusted for marital status, educational background (as linear term), residence, economic income and BMI.

### Association between 4DL and risk of disease-accompanied mortality

We found the protective effect of 4DL on the risk of disease-accompanied mortality. As shown in Table 3, the fully adjusted models suggested that compared with those with lower 4DL scores, the individuals with 3-4 had a significantly reduced risk of mortality accompanied by hypertension, heart disease, cerebrovascular disease, respiratory disease and dementia. Compared with 0 4DL score group, adhering to 3-4 4DL can reduce up to 36% risk of mortality accompanied by hypertension [HR: 0.64 (0.50-0.83), P<0.01], 42% risk of mortality accompanied by respiratory disease [HR: 0.58 (0.45-0.76), P<0.01] and 41% risk of mortality accompanied by dementia [HR: 0.59 (0.40-0.87), P<0.01]. However, the analyses did not show a similar protective effect on mortality accompanied by diabetes, cancer, gastric or duodenal ulcer and Parkinson’s disease (Supplementary Table 1).

**Table 3:** Multivariable-adjusted HRs (95% CIs) for disease-accompanied mortality by 4DL score among Chinese older people.

**4DL score**	**Participants**	**Death**		**Adjusted HR (95% CI)**	**P value**
Hypertension-accompanied mortality*			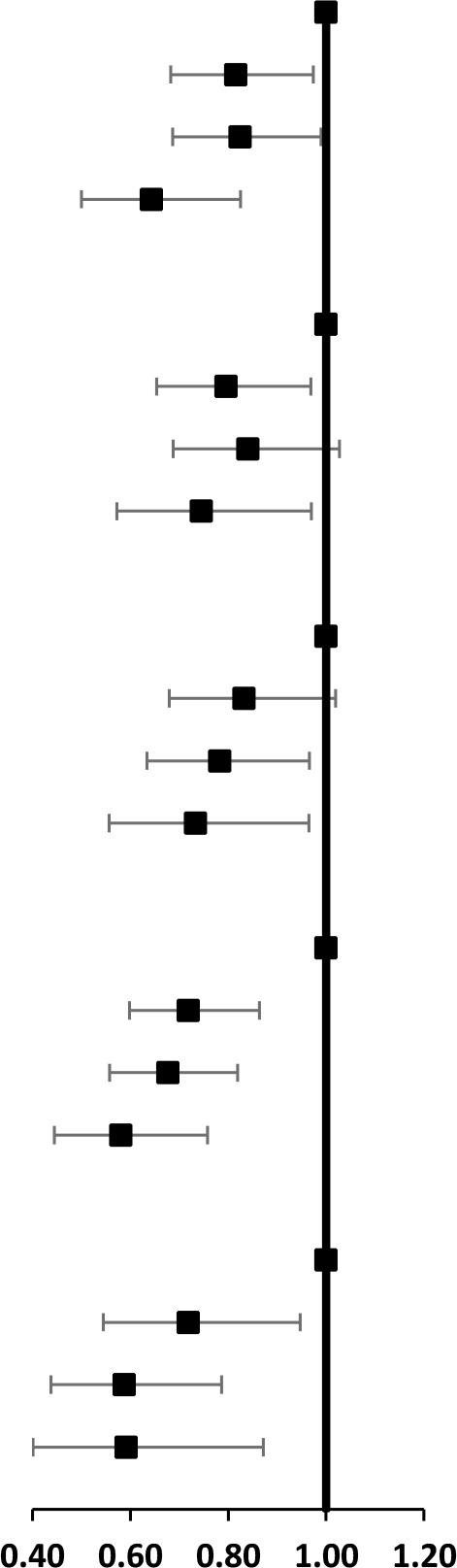		
0	1,497	170		1.00 (reference)	
1	6,155	560		0.82 (0.68-0.97)	0.02
2	4,622	451		0.82 (0.69-0.99)	0.04
3-4	1,350	104		0.64 (0.50-0.83)	<0.01
**HD-accompanied mortality**					
0	1,641	138			
1	6,789	453		0.80 (0.65-0.97)	0.02
2	5,035	379		0.84 (0.69-1.03)	0.09
3-4	1,485	101		0.75 (0.57-0.97)	0.03
**CVD-accompanied mortality**					
0	1,649	128			
1	6,878	457		0.83 (0.68-1.02)	0.08
2	5,218	333		0.78 (0.63-0.97)	0.02
3-4	1,604	92		0.73 (0.56-0.97)	0.03
**RD-accompanied mortality**					
0	1,543	166			
1	6,622	454		0.72 (0.60-0.86)	<0.01
2	5,044	336		0.68 (0.56-0.82)	<0.01
3-4	1,568	87		0.58 (0.45-0.76)	<0.01
**dementia-accompanied mortality**					
0	1,728	69			
1	7,166	253		0.72 (0.55-0.95)	0.02
2	5,467	158		0.59 (0.44-0.79)	0.00
3-4	1,689	44		0.59 (0.40-0.87)	0.01
				

*Cox model 2 were applied, with adjustment for sex, age (as a linear term), marital status, educational background (as a linear term), residence, economic income and BMI. *When examining the association between 4DL and mortality accompanied by hypertension, we excluded participants with physician-diagnosed hypertension at baseline to reduce the possibility of reverse causality. We adopted the same measure when examining other disease-accompanied mortality.

Abbreviations: HD: heart disease; CVD: cerebrovascular disease; RD: respiratory disease.

### Subgroup analyses and sensitivity analyses

**Figure 1:**
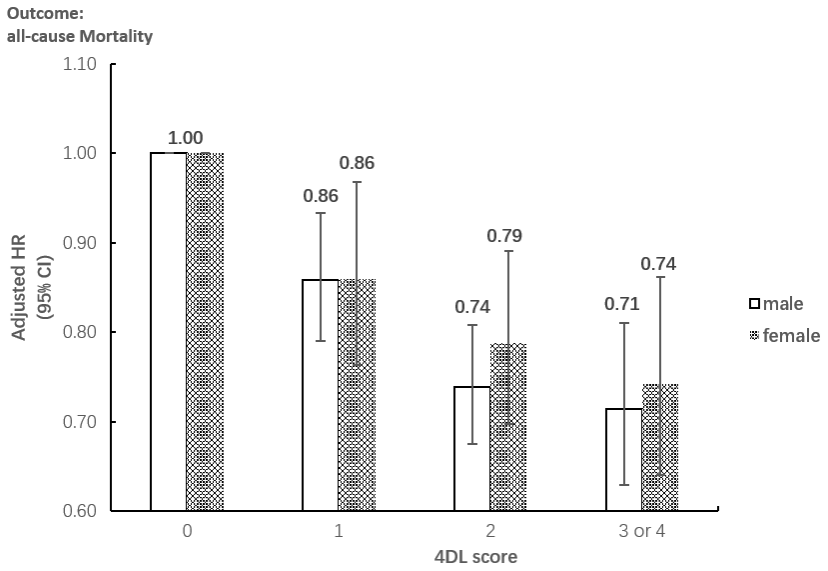
**Subgroup analysis between the association between 4DL and all-cause Mortality by sex.** Data are represented as hazard ratios with 95% confidential interval.

**Figure 2:**
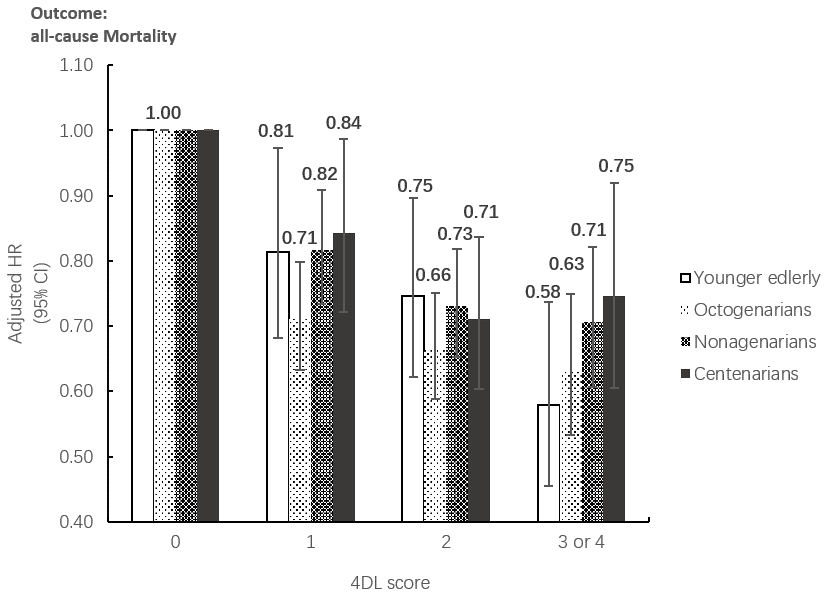
**Subgroup analysis between the association between 4DL and all-cause Mortality by age groups.** Data are represented as hazard ratios with 95% confidential interval.

The results of subgroup analyses on age and sex both remained significant for the associations between 4DL and all-cause mortality. Figure 1 presents that overall, the protective effect of 4DL on men was marginally more substantial than women. The study cohort selected participants with predefined sex and age, divided age groups into four: younger elderly (aged 65-79 years), octogenarians (aged 80-89 years), nonagenarians (aged 90-99 years), and centenarians (aged ≥100 years). We observed a similar protective effect in each subgroup (Figure 2). Compared with centenarians scored 0, centenarians with 3-4 4DL scores have a significantly reduced mortality risk [HR: 0.75 (0.61-0.92), P<0.01]. While among the younger elderly, the protective effect of adhering to 3-4 4DL was 17% lower [HR: 0.58 (0.45-0.74), P<0.01]. We performed two sensitivity analyses (SA) to evaluate the robustness of the association. Compared with the original analyses, the sensitivity analyses of SA1 and SA2 did not substantially alter the risk estimates (Supplementary Table 2).

## DISCUSSION

This study examines the association between 4DL and risk of mortality, including all-cause mortality and disease-accompanied mortality among the older population from different age groups in China. We found a significant relationship between adhering to 4DL and reduced all-cause mortality and mortality accompanied by hypertension, heart disease, cerebrovascular disease, respiratory disease and dementia. Our findings highlight the need to promote 4DL to reduce mortality risk, especially during the early aging period.

The similar mortality reduction by adhering to 4DL in Chinese older population corresponds to the results shown in western populations. A systematic review of 25 studies indicated that adhering to 4DL reduced 33% to 71% all-cause mortality risk [6]. The difference among these high-quality studies might result from various combinations of lifestyle patterns and different length of follow-up. In a meta-analysis of 15 cohort studies, Loef and Walach found that all-cause mortality risk reduced to 66% [95%CI: 0.58-0.73] in participants with four healthy lifestyle factors or more (alcohol consumption, non-smoking, healthy diet, and physical activity) [8]. Results from two prospective cohorts of about 64 000 Swedish adults aged 45–83 years showed that adhering to 4DL caused a reduction of all-cause mortality by between 52% to 58% compared with individuals with no or one healthy lifestyle factor [12]. Moreover, a study in the U.S. observed 37-54% reduction of all-cause mortality in group with 4 health lifestyle score compared with those with 0 [20]. Another study in Italy found that adhering to a different “4DL”(abstention from smoking; adherence to Mediterranean diet; physical activity; absence of abdominal obesity) could reduce 44%[95%CI: 0.39-0.81] of all-cause mortality risk compared with those have none or one “4DL” among elderly subjects [21]. Apart from the studies aiming at European and American populations, a prospective cohort study from China found that the hazard ratio of men aged over 40 years with 0 4DL is 2.92 compared with those with four healthy lifestyles [95%CI: 2.53-3.38] [22]. While in our study, we did not observe significant gender difference and suggested 4DL is a protective factor for both genders, which was consistent with research in America, Japan and the Netherlands [8].

Compared with former studies with more than 4 lifestyle factors, our study’s similar protective effects indicated that 4DL should be a scientific and sufficient pattern to evaluate lifestyles. Some researchers expanded 4DL and included BMI as the fifth lifestyle factor. In 2012, after combining the results from Asia, America and European population, Martin and Harald concluded that studies with BMI as the additional lifestyle behavior had the same results with those that adopted 4DL [8]. A study among the Chinese oldest-old (aged ≥80 years) suggested that the HR of all-cause mortality for the population in the upper tertile of 5-dimension protection score (including smoking, drinking, diet, exercise and weight) was 0.74 [95%CI: 0.70-0.77] compared with the lower tertile [5]. Other three studies including BMI or waist circumference as the fifth lifestyle factor also implied individuals accompanied with 4 to 5 healthy lifestyle factors had significantly lower all-cause mortality rate than those with 1 or less [23–25].

Several studies showed that adherence to healthy lifestyle reduced the risk of cardiovascular disease and heart disease [16, 18]. In Europeans and South Asians from the UK SABRE Study, a prospective analysis claimed that lack of adherence to four combined health behaviors was significantly related to a higher risk of incident coronary heart disease and CVD. Tsai et al. also found that the incidence of CVD decreased among adults with combined healthy lifestyle habits after conducting a meta-analysis of 22 eligible cohort studies [HR: 0.37 (0.31-0.43)] [26, 27]. Our research found the protective impact of 4DL on disease-accompanied mortality consistent with the former studies on different specific diseases. Furthermore, Zhang et al. found that combined 5-dimension healthy lifestyle factors, including waist-hip ratio, would prevent 21% colorectal cancer cases [95%CI: 0.04-0.36] [17]. In 2019, Bonaccio et al. examined the impact of healthy behaviors on cancer death. Significant protective effect among general adults was observed while the results among older population remain insignificant (n=365) [21]. A significant association was also not found between 4DL and cancer-accompanied mortality in our study, which may both due to the insufficient samples in this group (n=468). Though the former study showed that among men with type 2 diabetes, adhering to two or more healthy lifestyle factors would reduce more than 40% mortality risks compared with those adhering to one or none [28], our study has not observed significant result when examining diabetes-accompanied mortality (n=262).

Health status gap and health literacy difference could be possible mechanisms to explain the relationship between 4DL and reduced mortality risk. A Chinese randomized controlled trial found that adhering to recommended 4-dimension lifestyle behaviors (with specific quantitative index) resulted in significantly better health indicators (lower average systolic pressure, waist circumference, diastolic pressure, total cholesterol and fasting plasma glucose) at 24 months among the older population with a mean age of 70.53 years [9]. Indeed, our study discovered a lower risk of mortality accompanied by hypertension, heart disease, cerebrovascular disease, respiratory disease and dementia among a population with more healthy lifestyle factors. Better health status resulted from adhering to 4DL in the long-term follow-up could partly explain why mortality risk dropped [11, 29]. Former studies indicated interactions existed among the four factors in 4DL score. Jiang et al. concluded that diet quality could modify the association between alcohol consumption and health outcomes [30]. Another study found a significant association between adhering to smoking and inadequate physical activity in individuals older than 65 [31]. The synergy of the four factors might be explained by health literacy. Magnani et al. suggested that health literacy is associated with healthier lifestyle behaviors, which was consistent with overall findings presented before [32, 33]. The health and retirement study showed that better health literacy significantly contributed to perceived control of health among older adults [34]. With inadequate health literacy, physical health will worsen, resulting in higher mortality risk [35].

### Significance and limitations

In order to react to aging challenges, the low- and middle-income countries needed to collect more data and assemble solid evidence to compare with former studies in high-income countries and guide policy responses [36]. This study adds significant evidence about the protective effect of 4DL on the older population’s mortality risk in China. The CLHLS data had a large sample size and a long follow-up period, which allowed us to measure many potential confounders and draw a robust conclusion. With full-scale information from the CLHLS project, we also included adjustment for multiple sociodemographic confounders and sensitivity analyses to confirm the results’ robustness. Meanwhile, this study examined the risk of all-cause and disease-accompanied mortality risks influenced by 4DL among different age and sex groups, respectively. Giving the fact that the data collection among the elderly age group (>65 years old) could be quite challenging,, few studies investigated the impact of 4DL among different diseases or age groups. This study found that the mortality risk reduction caused by adhering to 3-4 4DL in the younger elderly (aged 65-79 years) was higher than those in other elder age groups, indicating maintaining 4DL at an earlier stage could reduce more mortality risk.

Nevertheless, this study also had several limitations. Firstly, when calculating the 4DL score, we found that few cases were scored at 4. Therefore, we combined the participants who had scores of 3-4 to have a stable category. Secondly, the 4DL score was calculated based on the baseline questionnaire, which did not consider possible changes during the analysis period. Thirdly, the lifestyle questionnaire was not adequate for calculating absolute levels of 4DL score. The precise physical activity level was unable to assess due to undefined difference between labor work and exercise. However, the self-reported exercise level based on participant’s comparison with individuals around him/her would be acceptable for ranking participants at a group level.

## CONCLUSIONS

Overall, our findings showed that maintaining 4DL were associated with lower all-cause mortality and mortality risk accompanied by hypertension, heart disease, cerebrovascular disease, respiratory disease and dementia. The positive effects of 4DL on longevity should be acknowledged in China’s older population, especially for younger elderly (aged 65-79 years).

## MATERIALS AND METHODS

### Study population and design

The CLHLS project has collected the older population’s full-scale information (aged>65 years) in China. The participants were selected randomly from half of the cities and counties in 22 provinces which covered 85% of the Chinese population. The CLHLS adopted a targeted random-sample design to draw an equal proportion of participants in different age and sex groups [37]. A structured questionnaire was adopted to collect the full-scale information of older population by a trained interviewer. The detailed description of the CLHLS project was discussed by former research [19]. Briefly, lifestyle information collected included food consumption frequency, smoking status, alcohol intake and physical activity level. The ethical approval of the CLHLS project was acquired from the Biomedical Ethics Committee of Peking University. All participants or their legal representatives signed written consent forms to participate in the baseline and follow-up survey.

In this study, we chose the fifth longitudinal wave of the CLHLS project, which included 16,954 participants enrolled in 2008 and followed up in 2011, 2014 and 2018 successively. Supplementary Figure 1 showed the exclusion process of participants. In total, this study included 16,280 participants and 27.0% were lost to follow up in 2018.

### Healthy lifestyle score

We adopted a modified 4DL to evaluate lifestyle factors, composed of dietary intake, smoking status, alcohol consumption and physical activity level. The 4DL score ranging from 0 to 4 points generated from the number of lifestyle recommendations that participant adhered to: (1) belonging to the low risk group of diet; (2) never smoking; (3) consuming alcohol without excessive intake; (4) regular exercises.

To assess the risk of dietary intake, we modified Mediterranean diet according to the Mediterranean diet Pyramid. It is a widely reported model of a healthy eating pattern composed of 9 components, including alcohol consumption; cereals; fresh fruits and nuts; fresh vegetables; legumes; a high ratio of monounsaturated to saturated fat; fish; dairy products and eggs; meat and meat products [38, 39]. Giving that cereals are commonly consumed in China (99.26% of the whole participants has reported consuming cereals), we did not include grains into the modified Mediterranean diet score. Moderate alcohol intake was also not included because we listed it as one of the other lifestyle factors and analyzed the effect of alcohol consumption separately. Therefore, the modified Mediterranean diet score was calculated from the remaining 7 components, and the recommended range was in Supplementary Table 3 [38]. If a person consumed certain food within the recommended range, we coded this variable as 1. We sum all 7 dichotomous variables to generate diet score, and it varied from 0 to 7. Individuals were considered to be at low risk if their diet score was more than 4.

### Outcomes

The outcomes were all-cause mortality and disease-accompanied mortality, including hypertension-accompanied mortality, diabetes-accompanied mortality, heart disease-accompanied mortality, cerebrovascular disease-accompanied mortality, respiratory disease-accompanied mortality, and dementia-accompanied mortality. The accompanying disease classification was based on the 10th revision of the International Statistical Classification (ICD-10) of Diseases, Injuries, and Causes of Death.

### Assessment of covariates

We classified “Marital status” as “not in marriage” (never married, widowed or divorced) and “in marriage” (currently married). “Residence” was categorised as urban (city or town residence) or rural (countryside residence). According to the question “Compared with other locals, how do you think about your economic position”, “Economic income” was categorised as “high”, “medium” or “low”. BMI was calculated as body weight (kg) divided by squared body height (m^2^). Overall, fewer than 2% of the data were missing for any single variable, and we adopted Mean value imputation methods to correct missing data in BMI and total schooling years.

### Statistical analysis

Based on the living status information, we divided the participant into three groups, including decedents (58.8%), survivors (14.2%) and participants who were lost to follow up (27.0%). The survival time for decedents was calculated according to how many years they have lived; the survival time for survivors was equal to total follow-up time; as for those who were unavailable to follow-up in 2011, 2014 or 2018, we performed censoring at 3 years, 6 years or 10 years. Cox proportional hazards models were adopted to estimate 4DL on all-cause mortality and mortality accompanied by a specific disease. We constructed two Cox models to correct the estimates for socioeconomic status: Model 1 adjusted age and sex at baseline; Model 2 additionally adjusted for marital status, educational background, residence, economic income and BMI. The results were shown in the tables and on the forest plot with estimated hazard ratios (HRs) and 95% confidential interval (95% CI).

### Subgroup analyses and sensitivity analyses

Subgroup analyses of different sex and age groups were also conducted, respectively. In sex-stratified analyses, the model was adjusted for age (as a linear term), educational background (as a linear term), marital status, economic income, residence, and BMI. To minimize potential bias resulting from subclinical conditions, we performed two sensitivity analyses (SA). SA1 excluded participants whose survival outcomes occurred in the first 3 years of follow-up; SA2 additionally excluded participants with physician-diagnosed diseases, including cancer, heart disease, cerebrovascular disease, Parkinson’s disease or dementia at baseline.

The criterion for statistical significance was determined via a two-sided test at a P-value <0.05. We used Stata version 15.0 and SPSS version 18.0 to conduct statistical analyses.

## Supplementary Material

Supplementary Figure 1

Supplementary Tables
