# Supplementary figures and images for: Association between 4-dimension lifestyle pattern and 10-year mortality risk in Chinese individuals older than 65: a population-based cohort study
Source: Aging (Albany NY). 2021 Mar 19;13(6):8835–48. doi: 10.18632/aging.202695 (PMC8034959; doi:10.18632/aging.202695)

SUPPLEMENTARY FIGURE

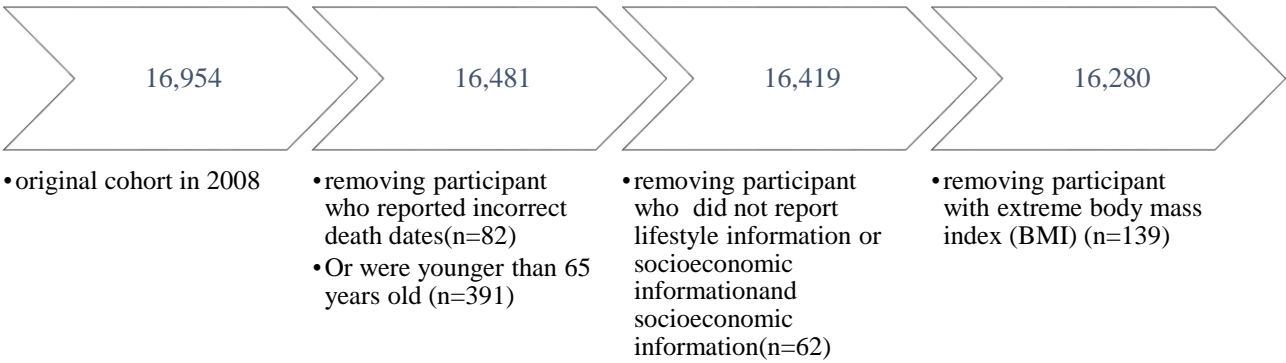

Supplementary Figure 1. Study sampling flowchart.

Supplement: Supplementary Figure 1 [file aging-13-202695-s001.pdf]
