# Supplementary material for: Association between 4-dimension lifestyle pattern and 10-year mortality risk in Chinese individuals older than 65: a population-based cohort study
Source: Aging (Albany NY). 2021 Mar 19;13(6):8835–48. doi: 10.18632/aging.202695 (PMC8034959; doi:10.18632/aging.202695)
Supplement: Supplementary Tables [file aging-13-202695-s002.pdf]

SUPPLEMENTARY TABLES

Supplementary Table 1. Multivariable-adjusted HRs (95% CIs) for disease-accompanied mortality by 4DL score among Chinese older people.

4DL score	Participants	Death	Adjusted HR (95% CI)	P value
**Diabetes-accompanied mortality**				
0	1,714	23	1.00 (reference)	
1	7,142	125	1.34 (0.85-2.12)	0.20
2	5,395	89	1.07 (0.67-1.71)	0.78
3-4	1,635	25	0.84 (0.47-1.50)	0.56
**Cancer-accompanied mortality**				
0	1,744	61	1.00 (reference)	
1	7,257	171	0.86 (0.64-1.16)	0.32
2	5,515	177	1.06 (0.78-1.43)	0.72
3-4	1,691	59	1.04 (0.72-1.51)	0.83
**Gastric or duodenal ulcer-accompanied mortality**				
0	1,669	30		
1	6,985	101	0.73 (0.48-1.11)	0.14
2	5,296	88	0.88 (0.58-1.35)	0.57
3-4	1,623	21	0.79 (0.45-1.40)	0.43
**Parkinson's disease-accompanied mortality**				
0	1,746	10		
1	7,251	38	1.01 (0.49-2.06)	0.98
2	5,523	23	0.79 (0.37-1.69)	0.55
3-4	1,697	10	1.20 (0.49-2.95)	0.69

*Cox model 2 were applied, with adjustment for sex, age (as a linear term), marital status, educational background (as a linear term), residence, economic income and BMI. *When examining the association between 4DL and mortality accompanied by diabetes, we excluded participants with physician-diagnosed diabetes at baseline to reduce the possibility of reverse causality. We adopted the same measure when examining other disease-accompanied mortality.

Supplementary Table 2. Sensitivity analysis between the association of 4DL and all-cause mortality.

4DL score	Participants	Death	Adjusted HR (95% CI)	P value
SA1				
0	1,714	23	1.00 (reference)	
1	7,142	125	1.34 (0.85-2.12)	0.20
2	5,395	89	1.07 (0.67-1.71)	0.78
3-4	1,635	25	0.84 (0.47-1.50)	0.56
**SA2**				
0	1,744	61	1.00 (reference)	
1	7,257	171	0.86 (0.64-1.16)	0.32
2	5,515	177	1.06 (0.78-1.43)	0.72
3-4	1,691	59	1.04 (0.72-1.51)	0.83

*SA1 was further excluded participants whose survival outcomes occurred in the first 3 years of follow-up; SA2 was further excluded participants with physician-diagnosed disease, including cancer, heart disease, cerebrovascular disease, dementia or Parkinson's disease at baseline.

Supplementary Table 3. The recommended range of the modified mediterranean diet.

Components	Recommended range (Dichotomous value=1)
Fresh fruits and nuts	Almost everyday
Fresh vegetables	Almost everyday
High ratio of monounsaturated to saturated fat	Preference to vegetable grease
Legumes	Not every day, but at least once per week
Fish	Not every day, but at least once per week
Dairy products and eggs	Not every day, but at least once per week
Meat and meat products	Once per week or less
